# Systems analysis of circadian time-dependent neuronal epidermal growth factor receptor signaling

**DOI:** 10.1186/gb-2006-7-6-r48

**Published:** 2006-06-19

**Authors:** Daniel E Zak, Haiping Hao, Rajanikanth Vadigepalli, Gregory M Miller, Babatunde A Ogunnaike, James S Schwaber

**Affiliations:** 1 Daniel Baugh Institute for Functional Genomics and Computational Biology, Department of Pathology, Thomas Jefferson University, Locust St, Philadelphia, PA, USA 19107; 2 Department of Chemical Engineering, University of Delaware, Academy St, Newark, DE, USA 19716

## Abstract

**Background:**

Identifying the gene regulatory networks governing physiological signal integration remains an important challenge in circadian biology. Epidermal growth factor receptor (EGFR) has been implicated in circadian function and is expressed in the suprachiasmatic nuclei (SCN), the core circadian pacemaker. The transcription networks downstream of EGFR in the SCN are unknown but, by analogy to other SCN inputs, we expect the response to EGFR activation to depend on circadian timing.

**Results:**

We have undertaken a systems-level analysis of EGFR circadian time-dependent signaling in the SCN. We collected gene-expression profiles to study how the SCN response to EGFR activation depends on circadian timing. Mixed-model analysis of variance (ANOVA) was employed to identify genes with circadian time-dependent EGFR regulation. The expression data were integrated with transcription-factor binding predictions through gene group enrichment analyses to generate robust hypotheses about transcription-factors responsible for the circadian phase-dependent EGFR responses.

**Conclusion:**

The analysis results suggest that the transcriptional response to EGFR signaling in the SCN may be partly mediated by established transcription-factors regulated via EGFR transription-factors (AP1, Ets1, C/EBP), transcription-factors involved in circadian clock entrainment (CREB), and by core clock transcription-factors (Rorα). Quantitative real-time PCR measurements of several transcription-factor expression levels support a model in which circadian time-dependent EGFR responses are partly achieved by circadian regulation of upstream signaling components. Our study suggests an important role for EGFR signaling in SCN function and provides an example for gaining physiological insights through systems-level analysis.

## Abstract 2

A systems level analysis of circadian time-dependent signaling via the epidermal growth factor receptor in the suprachiasmatic nucleus suggests several transcription factors that mediate the transcriptional response to epidermal growth factor receptor signaling.

## Background

The present work makes a systems level analysis of context-dependent signaling by the epidermal growth factor receptor (EGFR) in the suprachiasmatic nuclei (SCN). Circadian rhythms are driven by gene regulatory feedback networks [1], and in mammals the SCN comprise the master circadian clock [2]. SCN circadian rhythms are synchronized across SCN neurons [3], with the environment, and with the internal physiological state of the organism [4]. Importantly, the effects of phase modulating extracellular inputs to the SCN are regulated by the circadian clock itself and are thus 'gated' [5] or circadian time dependent. Biochemical correlates of light (for example, glutamate), for instance, have little effect during the circadian day, but cause phase delays in the early night, and phase advances in the late night [5]. The mechanisms behind circadian time dependent signaling in the SCN are largely unknown, but mechanisms that in other systems give rise to signaling specificity - cell-type specific responses to the activation of common signaling pathways (reviewed in [6,7]) - may be partly responsible. Phases of differential SCN signaling responsiveness cycle with circadian time, however, and thus components responsible for circadian modulation of signaling responses must also cycle with circadian time, rendering the SCN a particularly interesting and well-contained system for studying context-dependent signaling.

Recent studies suggest important roles for EGFR signaling in the regulation of circadian rhythms by the SCN. EGFR and EGFR ligands are expressed throughout the central nervous system and are involved in diverse developmental and homeostatic processes [8]. SCN expression of EGFR [9-11] and transforming growth factor alpha (TGF-alpha; an EGFR ligand) [9,10,12,13] have been reported. EGFR signaling has been implicated in the circadian regulation of locomotor activity [13] and grooming, exploring, and feeding behaviors [14]. EGFR activation has been found to induce Erk phosphorylation in the SCN [15]. Elevated TGF-alpha serum levels have been observed in cancer patients with dampened circadian activity rhythms [16]. Furthermore, roles have been suggested for EGFR signaling in clock regulation via retinal SCN inputs [13] and the intercellular synchronization of SCN rhythms [10]. Lastly, a microarray study of SCN circadian gene expression revealed rhythmic EGFR substrate expression [17]. The signaling pathways downstream of EGFR are uncharacterized in the SCN, but by analogy to other well-characterized SCN inputs [5], and studies in other systems of context-dependent EGFR signaling [18], we hypothesize that the EGFR signaling in the SCN is circadian time dependent.

In this work, a preliminary characterization of the transcriptional pathways underlying circadian time dependent EGFR signaling in the SCN is made. Factorial-designed microarray experiments [19] are combined with mixed-model analysis of variance (ANOVA) [20], enrichment analyses, and promoter bioinformatic techniques [21] to generate hypotheses about the transcription factors (TFs) regulating genes with circadian time dependent expression responses to EGFR activation. This work is consistent with others in which microarray analysis was combined with promoter analysis to generate hypotheses about the TFs regulating circadian gene expression [22], and expression responses to specific signaling pathways [23,24]. We extended the methods of these previous works by performing thorough microarray and promoter analyses and by seeking results that were both statistically significant and robust to variations in analysis parameters, following recommendations in [25]. We found strong support for circadian time dependent EGFR responses in the SCN, and quantitative real-time (qRT)-PCR measurements of a subset of implicated TFs revealed that circadian time dependent EGFR responses may be partly due to circadian modulation of upstream signaling pathways.

## Results and discussion

The objectives of the current study were to identify genes responsive to EGFR signaling in the SCN, to determine whether these responses are circadian time dependent, to identify the pathways and functions modulated by EGFR signaling in the SCN, and to make hypotheses about the regulators responsible for the EGFR responses. To these ends, a 2^2 ^factorial designed microarray experiment was performed in which the SCN responses to EGF treatment during the 'day' (8 hours after lights on) and 'night' (2 hours after lights off) were compared. Genes with expression levels regulated by EGFR signaling in a circadian time dependent manner were identified using mixed-model ANOVA. To generate hypotheses about the pathways and cell functions modulated by EGFR signaling in the SCN, we tested for enrichments of previously established circadian gene expression [17,22] and Gene Ontology (GO) terms in groups of EGF responsive genes. To generate hypotheses about the regulators underlying the circadian time dependent EGFR responses in the SCN, we tested for enrichment of TF binding predictions in the promoters of EGF responsive gene groups. Given that TF binding site databases are currently incomplete, with the number of known or predicted TFs greatly exceeding the number of well-characterized TF binding sites, and given that the quality and specificity of binding sites differs across databases and data sets, we sought consistent hypotheses by utilizing three complementary sources of TF binding predictions: the TRANSFAC^® ^database [26], predictions based on phylogenetic conservation [27], and genome-wide location analysis data [28,29]. By seeking consistent results from complementary data sources, we feel we overcome some of the limitations in relying on TF binding site predictions to infer regulatory networks, even though identifying the specific TFs acting at implicated binding sites remains an important challenge.

Our experiments and analyses provide evidence for circadian-time dependent EGFR responses that are relevant to circadian clock function. Additionally, we identified several TFs known to be downstream of EGFR signaling and generated hypotheses about their roles in regulating the responses.

### Topology of EGFR-responsive gene expression

**Figure 1:**
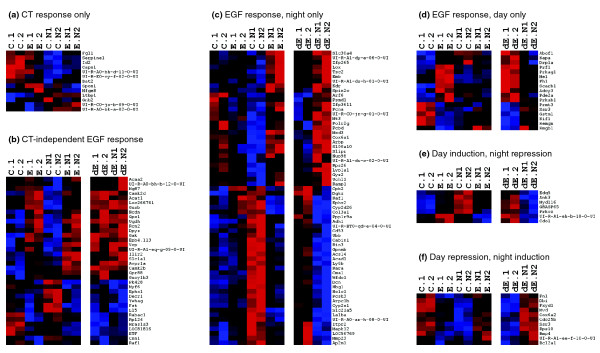
EGFR activation induces circadian time (CT) dependent and CT independent transcriptional programs in the SCN. Results for genes with expression changes detected at a False Discovery Rate (FDR) <2% (see Materials and methods). (**a**) Genes with expression levels modulated by CT but not EGF treatment. (**b**) Genes with expression responses to EGFR activation that were not CT-dependent. (**c**) Genes with expression levels modulated by EGFR activation at nighttime only. (**d**) Genes with expression levels modulated by EGFR activation during daytime only. (**e**) Genes up-regulated by EGFR activation during the circadian day and repressed at night. (**f**) Genes down-regulated by EGFR activation during the circadian day and induced at night. Blue and red shades represent negative and positive scaled log_2_-expression levels or expression differences, respectively. C.1 and C.2 represent daytime control rats whereas C.N1 and C.N2 represent nighttime control rats. E.1 and E.2 represent EGF-treated (20 nM, 1 hour) daytime rats while E.N1 and E.N2 represent EGF-treated nighttime rats. Log_2_-expression levels in these cases were scaled for each gene by first subtracting random components for each rat, and then subtracting the mean log_2_-expression level over all conditions. To facilitate comparisons between genes, these mean-zeroed expression levels were divided by their maximum absolute value. dE.1 and dE.2 represent scaled EGF-induced log-expression differences in daytime rats while dE.N1 and dE.N2 represent scaled EGF-induced log-expression differences in nighttime rats. To facilitate comparisons between genes, these expression differences were divided by their maximum absolute value. Genes are represented by gene symbols, except in cases where annotation was not available and clone IDs are given instead. Images were created using the free Treeview program [62]. Additional data file 1 displays the relativeanimal-animal variability in the expression responses for a selected subset of genes.

At a false discovery rate (FDR) of 2%, approximately 10% of the genes on our microarrays were EGF responsive. Heatmaps showing the diversity of observed expression responses to EGFR activation are given in Figure 1. Interestingly, the majority (approximately 70%) of the EGF-responsive genes had EGF responses that depended in some way on the circadian time at which the EGF treatment was made. These were identified in the mixed model ANOVA as those with statistically significant EGF:circadian time (EGF:CT) interaction effects on gene expression levels (see Materials and methods). While the genes on our microarrays are not necessarily representative of the entire genome, these results suggest that: (1) the SCN is transcriptionally responsive to EGFR activation, and (2) the pathways by which EGFR activation leads to gene regulation are modulated by circadian time. Focusing specifically on genes most strongly regulated by EGFR activation revealed several involved in EGFR responses in other systems. Subsets of these are shown in Additional data file 1 and are discussed in Additional file 6. *P *values for EGF effects and EGF:CT interactions for all genes meeting quality control criteria are given in Additional data file 3.

### EGFR modulation of circadian cycling genes in the SCN

To determine whether the EGF responsive genes we identified have a role in core clock function, we compared them to previously established circadian cycling genes in the SCN [17,22]. Specifically, we tested whether circadian-regulated genes were over-represented in our EGF-responsive gene groups compared to random gene groups of the same size. We did not find statistically significant enrichment for circadian genes identified in [22] for any of our EGF responsive gene groups. For the rhythmic SCN genes identified in [17], however, we observed enrichments in select EGF responsive subsets (Table 1, marked 'Circ.'). Using a gene significance cutoff of 10% FDR, the overall EGF responsive gene set was enriched for circadian genes (*p*_ENRICH _< 0.05, *p*_M_^(LOCAL) ^< 0.05), containing 40% of the circadian genes on the array. Similarly, the genes with CT-dependent EGF responses were enriched for circadian genes (*p*_ENRICH _< 0.05, *p*_M_^(LOCAL) ^< 0.05). These results suggest that EGFR in the SCN modulates bioprocesses that are relevant to circadian clock function. That we found enrichments for one set of circadian genes and not another is not troublesome given the substantial differences between these lists [30]. *P *values for EGF effects and EGF:CT interactions for the circadian cycling genes identified in [17] that were present on our arrays are given in Additional data file 4.

### EGFR-responsive pathways and functions

Hypotheses concerning differentially regulated processes/functions by EGFR in the SCN were derived from tests for statistically significant (*p*_ENRICH_^(FDR) ^< 0.11) and robust (*p*_M_^(LOCAL) ^< 0.06, *p*_M_^(GLOBAL) ^< 0.1) GO term enrichments in the EGF-regulated gene groups. Results for gene groups defined at FDR <1% are shown in Table 1 (marked 'GO'). Over 20% of the genes on the array annotated with 'protein serine/threonine kinase activity' were EGF-responsive in some manner, a 2.8-fold enrichment over random groups (*p*_ENRICH _< 0.002) that was also robust (*p*_M_^(LOCAL) ^< 0.02, *p*_M_^(GLOBAL) ^< 0.07). The genes are involved in diverse pathways, including *PKCz*, *PKAβ1*, *Kdr*, two isoforms of *CamKII *(*Camk2b *and *Camk2d*), *MAPK12*, and *Raf-1*. Similarly, 28% of the genes annotated with 'cell differentiation' on the array were EGF-responsive, a 3.4-fold enrichment over random (*p*_ENRICH _< 0.003) that was robust (*p*_M_^(LOCAL) ^< 0.02, *p*_M_^(GLOBAL) ^< 0.06). Furthermore, genes with EGF:CT interactions were robustly significantly enriched for 'cell differentiation', while genes with EGF responses independent of circadian time were significantly enriched for 'protein serine/threonine kinase activity'. These results suggest separate regulation of these processes in the SCN.

### EGFR-mediated transcriptional regulation

#### TF binding site family predictions from MATCH/TRANSFAC Pro using PAINT

We first utilized MATCH/TRANSFAC Pro predictions of TF family binding in rat gene promoters as accessed through the bioinformatics tool PAINT [21]. Robust statistically significant enrichments obtained using PAINT were defined as those for which *p*_ENRICH _< 0.1 and *p*_M_^(LOCAL) ^< 0.06. Results for the gene groups defined at FDR <2% are shown in Table 1 (marked 'PAINT').

CREB family binding sites were robustly significantly enriched in the superset of EGF-responsive genes (*p*_ENRICH _< 0.005, *p*_M_^(LOCAL) ^< 0.005, *p*_M_^(GLOBAL) ^< 0.1), and genes with EGF:CT interactions (*p*_ENRICH _< 0.01, *p*_M_^(LOCAL) ^< 0.01). CREB is an established EGFR signaling target in neurons [31] and plays a critical role in SCN core clock gene network regulation by light [32]. Our results, showing enrichment of predicted CREB binding sites in promoters of genes with CT dependent EGFR responses, are consistent with a model in which the circadian clock regulates regulators of CREB activity [33]. Lastly, the role for CREB in circadian rhythm modulation by EGFR signaling (predicted presently) is supported by previous work in which intraperitoneal EGF injections reduced phosphorylated CREB levels in the esophagus and phase shifted esophageal circadian DNA synthesis rhythms [34].

Genes with circadian time dependent EGF responses were robustly significantly enriched for binding site family predictions of other EGFR target TFs. These include: CRE-BP1 [35], with CRE-BP1:c-Jun family sites enriched at *p*_ENRICH _< 0.02 and *p*_M_^(LOCAL) ^< 0.02); and c-Ets1 [36], with c-Ets-1/68 family sites enriched at *p*_ENRICH _< 0.1 and *p*_M_^(LOCAL) ^< 0.06. Lastly, 50% of the genes on our arrays with CREBATF family binding sites had significant EGF:CT interactions, a highly significant (*p*_ENRICH _< 0.001) 5.7-fold enrichment over random that was also locally robust (*p*_M_^(LOCAL) ^< 0.02).

#### TF binding site predictions based on phylogenetic conservation

To supplement the results obtained using PAINT, we tested for robust statistically significant enrichments of TF binding site predictions based on phylogenetic conservation from [27]. Since these conserved sites were reported for human genes, we mapped them to the rat genes on our arrays using Homologene [37]. Given the large number of TF binding site predictions obtained using this method, robust statistically significant enrichments were defined using more stringent cutoffs than for PAINT (*p*_ENRICH _< 0.03 and *p*_M_^(LOCAL) ^< 0.06). Results for the gene groups defined at FDR <2% are shown in Table 1 (marked 'CONS').

The predicted TF binding site most significantly and robustly enriched was V$RORA1_01 (vertebrate RORα1 matrix 1), which was enriched in the superset of EGF-responsive genes (*p*_ENRICH _< 1 × 10^-4^, *p*_M_^(LOCAL) ^< 5 × 10^-4^, *p*_M_^(GLOBAL) ^< 5 × 10^-3^), genes with EGF:CT interactions (*p*_ENRICH _< 5 × 10^-4^, *p*_M_^(LOCAL) ^< 5 × 10^-3^, *p*_M_^(GLOBAL) ^< 5 × 10^-3^), and genes responsive to EGF only during the night (*p*_ENRICH _< 2 × 10^-3^, *p*_M_^(LOCAL) ^< 0.01, *p*_M_^(GLOBAL) ^< 0.05). Effectively, significant enrichment for V$RORA1_01 sites was independent of the significance cutoff used to define gene groups and even the method used to normalize the array data (of those considered). Although the TFs that bind RORα1 sites are not established targets of EGFR signaling, two of them (Rorα and Rev-erb-alpha) are essential components of the circadian clock gene network in the SCN [22,38,39]. Involvement of Rorα binding sites in the circadian time dependent transcriptional response of the SCN to EGFR may provide a direct link between EGFR signaling in the SCN and the core clock.

Binding site predictions for CCAAT/enhancer binding protein (C/EBP) TFs, some of which are known targets of EGFR signaling in other systems [40,41], were also robustly significantly enriched in the promoters of EGF-responsive gene groups. V$CEBP_Q2 and V$CEBP_Q3, respective binding sites for C/EBPα and the C/EBP family broadly, were enriched in the EGF-responsive superset, genes with EGF:CT interactions, and genes regulated by EGF during the night only; whereas V$CEBPGAMMA_Q6 was robustly significantly enriched in the EGF-responsive superset and genes with circadian time independent EGF responses. These results suggest differential utilization of C/EBP TFs downstream of EGFR signaling in the SCN to achieve circadian time dependent and circadian time independent responses. Recent work showing core clock gene induction by C/EBPα in other systems [42] supports a role of C/EBPα in circadian signaling.

Many enrichment results obtained using phylogenetically conserved binding site predictions [27] corroborated those from PAINT, strengthening regulatory hypotheses. Robust and significant enrichments for c-Ets1 binding sites were found in EGF:CT interaction genes: for PAINT, c-Ets-1/68 family sites were enriched, while phylogenetic conservation predictions yielded V$CETS1P54_01 enrichment. Using PAINT, CRE-BP1:c-Jun family sites were enriched in EGF:CT interaction genes, while phylogenetic conservation predictions yielded enrichment for V$CREBP1_Q2 in that same gene group and robust significant enrichment for V$CREBP1_01 in the genes responsive to EGF during the night only. Enrichment results obtained using PAINT and phylogenetic conservation jointly support a hypothesis for the involvement of c-Jun, a component of the EGFR activated TF AP1 [43], in the SCN EGFR response, given the enrichments of CRE-BP1:c-Jun family sites and the AP1 consensus site (V$AP1_C) in the EGF:CT interaction genes obtained using those methods, respectively. Finally, significant enrichment of specific phylogenetically conserved CREB binding sites (V$CREB_Q2, V$CREB_Q4, and V$CREB_Q4_01) was found for genes with EGF:CT interactions that were responsive both during the day and night - approximately 50% of the genes in this group had either the V$CREB_Q4 or V$CREB_Q4_01 in their promoters. Since this gene group is a subset of the gene groups for which CREB family enrichments were observed using PAINT, these enrichments provide additional support for CREB involvement.

#### TF binding predictions from protein-DNA interaction data

As a final step in generating regulatory hypotheses, we tested for experimentally established TF promoter binding enrichment in the EGF-regulated gene groups. The available mammalian system-wide protein-DNA interaction data are limited, but the location analysis studies in [28,29] provide genome-wide promoter binding predictions for CREB and three hepatocyte nuclear factor (HNF) family members in human non-neuronal cells, respectively. To utilize these data in our study, we mapped the human gene data to the rat genes on our arrays using Homologene. Enrichment results for gene groups defined at FDR <2% are shown in Table 1 (marked 'ChIP').

In spite of the fact that the protein-DNA interaction data are for non-neuronal human cells, moderate enrichments were observed for HNF1-alpha and CREB in our gene groups, although neither were robust to the significance threshold for gene expression effects (*p*_M_^(LOCAL) ^> 0.15 for both TFs) or variations in the normalization method (*p*_M_^(GLOBAL) ^> 0.20 for both TFs). Significant enrichment (*p*_ENRICH _< 0.04) of HNF1-alpha, an EGF-regulated TF in some systems [44], was observed in the genes with circadian time dependent EGF responses. Fifty percent of the genes with EGF:CT interactions were bound by CREB in at least one condition in the data from [28], a 1.7-fold enrichment over random that is significant at a low level (*p*_ENRICH _< 0.12). Taken with the robustly statistically significant CREB enrichments obtained using PAINT and phylogenetic conservation TF binding site predictions, this result provides additional support for the hypothesis of CREB involvement in the circadian time dependent SCN EGFR response.

### qRT-PCR validation of TFs implicated by gene group enrichment analyses

**Figure 2:**
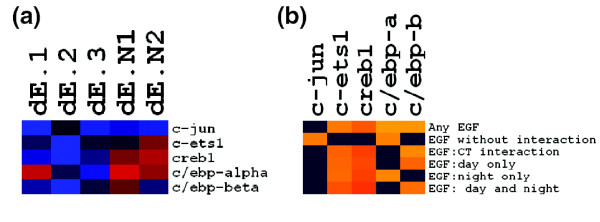
TF transcriptional responses to SCN EGFR activation and their expression correlations with target gene groups. (**a**) Gene expression responses to EGFR activation of five TFs implicated by the gene group enrichment (qRT-PCR). *c-Jun *is consistently down-regulated during both day and night, *c-Ets1 *and *Creb1 *are both down-regulated during the day and up-regulated during the night, *C/EBPα *is consistently up-regulated during the night only, and *C/EBPβ *is consistently down-regulated during the day only. Red and blue shades represent positive and negative changes in expression, respectively. dE.1, dE.2, and dE.3 represent scaled normalized -ΔCt values (approximate log_2 _expression levels, see Materials and methods) in daytime rats while dE.N1 and dE.N2 represent scaled -ΔCt values in nighttime rats. To facilitate comparisons between genes, expression differences were divided by their maximum absolute values. Additional data file 2 displays the relative animal-animal variability in the expression responses. (**b**) Statistically significant (*p *< 0.01) average absolute Pearson correlations between scaled log_2 _TF expression levels (qRT-PCR) and scaled log_2 _expression levels of EGF responsive genes (microarray). *Creb1 *expression was strongly correlated with expression profiles of putative circadian time dependent target gene groups whereas *c-Ets1 *expression was more weakly, but nevertheless significantly, correlated with those gene groups. *c-Jun *was predicted to regulate target genes in a circadian time dependent manner but has a circadian time independent expression response that is significantly correlated with the circadian time independent gene group. *C/EBPβ *expression was significantly correlated with putative daytime C/EBP target genes while *C/EBPα *expression was significantly correlated with putative C/EBP target nighttime responsive genes. Black squares indicate the absence of statistically significant correlations whereas orange squares indicate the presence of statistically significant correlations. Correlation strength is represented by color intensity, with the lowest significant average absolute correlation being 0.5 (between *C/EBPα *and the overall EGF responsive gene set) and the highest significant average absolute correlation being 0.9 (between *Creb1 *and the genes responsive to EGF during the day and the night). Images for (a) and (b) were created using the free program Treeview [62].

As a preliminary experimental validation of the gene group enrichment analysis results, we tested using qRT-PCR for differential expression of several TFs with robustly enriched binding sites. Given the possibility of post-transcriptional TF regulation, however, negative results do not necessarily invalidate the enrichment results. Based on the robust significant enrichments for binding site predictions, *Creb1*, *c-Ets1*, *c-Jun*, C/EBPα, C/EBPβ, C/EBPγ, *Ror α *and *Ror β *were selected for validation. Results of the qRT-PCR validation experiments are discussed below and shown in Figure 2 and Additional data file 2.

*c-Jun *was weakly but consistently down-regulated (*p*^EGF ^≤ 0.06) in response to EGF treatment during the day and the night. *Creb1 *and *c-Ets1*, however, were down-regulated in response to EGF during the day and up-regulated by EGF during the night (both 1.5-fold). These responses were statistically significant (*p*^EGF:CT ^< 5 × 10^-5 ^for *Creb1 *and *p*^EGF:CT ^< 5 × 10^-3 ^for *c-Ets1*). *C/EBPα *was consistently up-regulated during the circadian night only (*p*^EGF:CT ^< 0.05, 4-fold induction), while *C/EBPβ *was consistently down-regulated during the circadian day only (*p*^EGF:CT ^< 5 × 10^-3^, 3-fold repression). Although we did not detect *C/EBPγ *expression in one of our daytime EGF-treated samples, we found it to be weakly repressed by EGFR signaling in a CT-independent manner (*p*^EGF ^< 0.05). We did not observe any statistically significant effects on *Rorα *and *Rorβ *expression. Interestingly, significant CT effects on expression (in the absence of EGF) were not observed for any of the TFs considered. It is possible that transcripts for these TFs do cycle with circadian time, but were not detected as such because of our choice of circadian time points or because the changes are small relative to the animal-animal variability. In spite of these possibilities, we will base our subsequent regulatory hypotheses on our inability to detect circadian expression changes and will leave further verification for future studies. We also note that we observed *Creb1 *expression responses even though CREB is generally considered a constitutive TF [45]. Although rare, there are examples of *Creb1 *gene expression changes in response to extracellular stimuli in neurons [46]. The novel changes observed in the current study warrant further investigation.

Previous studies have suggested that expression profile correlations may be indicative of functional regulatory relationships between TFs and their target genes [47]. While we have demonstrated that gene dynamics may lead to more complex relationships between TF and target expression patterns in some conditions [48], we nevertheless undertook an analysis to test for significant correlations between the selected TFs and the EGF responsive gene groups. Specifically, we tested whether the average absolute value of the correlations between TF expression profiles and the EGF responsive gene groups were greater than the correlations between the TFs and random gene groups of the same size. We observed statistically significant (*p *< 0.01) correlations between the implicated TFs and the EGF responsive gene groups in the SCN for all TFs considered except *C/EBPγ *(Figure 2b). As discussed above, we found multiple lines of evidence supporting a role for CREB in regulating the CT-dependent EGF responses in the SCN. Further support for this relationship was given by significant correlations between the *Creb1 *expression profile and the expression profiles of genes in the gene groups enriched for CREB-related binding sites (*p *= 5 × 10^-4 ^in all cases). We also observed significant correlation between the *c-Ets1 *expression profile and the profiles of the CT-dependent EGF responsive gene group that was enriched for c-Ets1 related binding sites (*p *= 5 × 10^-4^); between the *C/EBPα *expression profile and the profiles of the gene group that was EGF responsive during the night only and was enriched for C/EBP related binding sites (*p *= 5 × 10^-4^); and between the *C/EBPβ *expression profile and the profiles of the gene group that was EGF responsive during the day only that was enriched for C/EBP related binding sites (*p *= 5 × 10^-4^). It must be noted that significant correlations between these TFs and gene groups that were not enriched for their binding sites were also observed, demonstrating potential limitations in relying solely on expression profile correlations to link TFs to their targets [48]. Interestingly, the correlation between *c-Jun *and the genes with CT-independent EGF responses was statistically significant (*p *= 5 × 10^-4^), even though this gene group was not enriched for c-Jun related binding sites. It is thus likely that CT-dependent post-transcriptional mechanisms are responsible for the CT-dependent target gene regulation that appears to involve this TF.

A plausible hypothesis to explain the putative circadian time dependent regulation of EGFR target genes by these TFs is that they are available to be regulated by EGFR signaling at some circadian times but not others. This mechanism for context-dependent regulation has been observed previously [7] and would be supported by strong circadian variation in TF mRNA levels. The qRT-PCR results for the TFs considered, showing no significant circadian variation in gene expression, do not support this hypothesis, and an alternative mechanism is required. The expression responses of *Creb1*, *c-Ets1, C/EBPα*, and *C/EBP β *to EGF treatment were themselves circadian time dependent, and it is thus possible that these expression changes partially account for the putative circadian time dependent regulation of target genes by these TFs. In this case, the circadian clock must modulate the upstream signaling pathways that lead to their gene regulation. *c-Jun *expression regulation by EGF at the mRNA level was circadian time independent and thus cannot account for circadian time dependent gene-expression regulation downstream of EGFR. Circadian time dependent post-transcriptional regulation of *c-Jun *activity or circadian time dependent regulation of *c-Jun *cofactors would be required for regulation of circadian time dependent EGFR responses.

**Figure 3:**
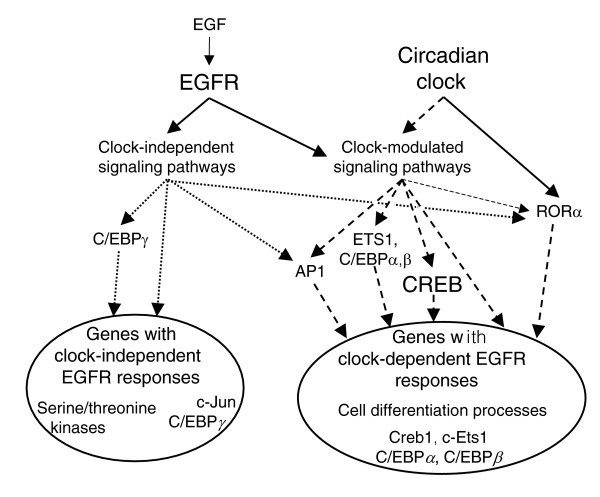
Hypothesized regulatory interactions that partially account for circadian time dependent EGFR transcriptional responses in the SCN. Modulation of the SCN gene expression response to EGFR activation (via EGF) by the circadian clock was investigated in the present study. We identified groups of genes with both CT-dependent and CT-independent expression responses, and these groups were enriched for specific cellular functions and the presence of specific TF binding sites in their promoters. Genes with CT-independent EGF responses were enriched for serine/threonine kinase activity and for C/EBPγ binding sites in their promoters. Given their CT-independent responses, and given that we observed weak CT-independent *C/EBP*γ expression responses to EGF, it is plausible that the EGFR-regulated signaling pathways responsible for their induction through C/EBPγ-dependent and -independent mechanisms function independently of the circadian clock. Genes with CT-dependent responses were enriched for involvement in cellular differentiation processes and the presence of c-Ets1, AP1, C/EBP, RORα, and CREB binding sites. Although RORα is a direct regulatory target of the circadian clock, we did not observe CT or EGFR expression responses for this gene and, thus, it may cause EGF induced CT-dependent gene regulation through post-transcriptional mechanisms. On the other hand, we did observe CT-dependent EGFR expression responses of *c-Ets1*, *Creb1*, *C/EBPα*, and *C/EBPβ*, constituting a mechanism by which these genes may cause CT-dependent expression responses of their target genes, and indicating that these TFs must be regulated by CT-dependent pathways. Interestingly, *c-Jun *EGFR expression responses were CT-independent, indicating that it must regulate CT-dependent expression responses through CT-dependent post-transcriptional mechanisms. Solid lines indicate direct interactions, dotted lines represent indirect CT-independent interactions, and dashed lines represent indirect CT-dependent interactions. CREB is emphasized given the strong support provided by multiple independent analyses for its involvement in the EGFR response.

A schematic summarizing all of the predicted regulatory interactions is provided in Figure 3.

## Conclusion

Our factorial-designed microarray experiments, mixed-model ANOVA, gene group enrichment analyses, meta-analyses, and qRT-PCR validations provide insight into the regulation of circadian time dependent EGFR signaling in the SCN. Even though the arrays that we used were relatively small in scale, the extensive functional annotation of the genes allowed us to perform gene group enrichment analyses from which regulatory hypotheses were derived. Several of the hypotheses are consistent across the different TF binding predictions utilized, giving us greater confidence that they provide clues to the underlying biology. By performing meta-analyses of our enrichment results, we were able to identify results that were robust to small variations in the significance thresholds and normalization procedures and, therefore, potentially more reflective of the underlying biological waves of regulation.

The extensive literature information about EGFR signaling in other systems allowed us to put many enrichment analysis results into appropriate context. The regulatory hypotheses we developed, based on our microarray experiments, GO information, several sources of TF binding predictions, qRT-PCR experiments, and the literature, are summarized in Figure 3. Interestingly, the two TF binding sites that were most strongly enriched in all of the analyses, CREB and RORA1, are very similar to the two significant binding sites identified in a previous promoter bioinformatics study of genes with circadian expression patterns in the SCN [22], providing additional evidence for a link between SCN EGFR signaling and the core circadian gene regulatory network. Our results support a functional role of EGFR signaling in the circadian clock, give insights into the mechanisms underlying functional input integration in the SCN, and provide a framework for further analysis of this important physiological process.

## Materials and methods

### Experimental design

**Figure 4:**
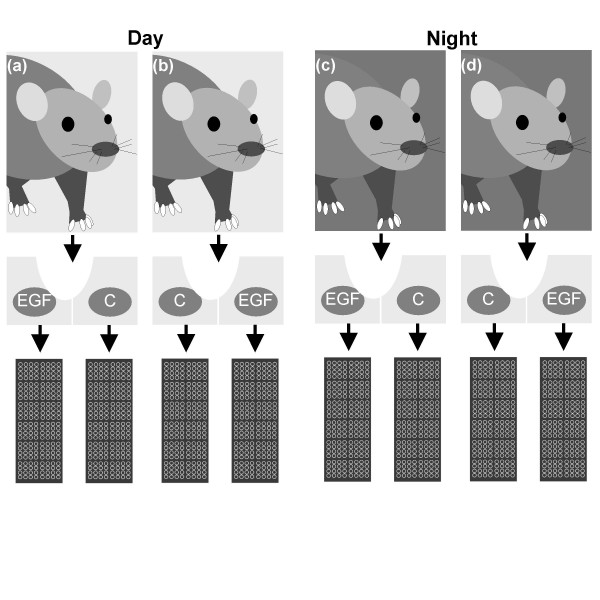
Experimental design. We used a total of four rats in the present microarray studies, two for the circadian day (8 hours after lights on, rats (a) and (b)), and two for the circadian night (2 hours after lights off, rats (c) and (d)). From each rat we obtained coronal slices that contained two SCN (left and right), separated by the third ventricle. Slices were separated along the third ventricle and placed in media containing EGF (20 nM) or control vehicle (C) for one hour. RNA for use with the microarrays was then extracted from SCN punches from the slices.

We investigated the difference in SCN gene expression between 'day' (8 hours after lights on) and 'night' (2 hours after lights off) following EGFR activation by EGF treatment. Circadian phase shifts induced by other stimuli have been reported at these time points [5], rendering them good candidates for interrogating EGFR-induced gene expression. Two SCN were obtained from each rat for EGF-treated and vehicle-treated samples. Pairing control and treated samples from the same rat permitted detection of EGF effects in the presence of substantial animal-to-animal variability. SCN from two rats were treated at each circadian time, yielding a total of eight biological samples. Since our goal was a preliminary characterization of EGFR response circadian time dependency, samples were hybridized to one microarray each. An experimental design schematic is given in Figure 4. A universal reference design was employed for the microarrays themselves [49].

### SCN sample preparation

Adult Sprague-Dawley rats (100 to 150 g) housed individually and entrained to 12:12 light-dark cycles (lights on at 6:00 AM and lights off at 6:00 PM) for at least two weeks were rapidly sacrificed between 10:00 AM and 12:00 PM for daytime treatments and between 4:00 PM and 6:00 PM for night treatments according to a protocol approved by TJU Institutional Animal Care and Use Committee. Brains were excised quickly, placed in ice-cold, oxygenated artificial cerebral spinal fluid (ACSF; 10 mM HEPES, pH 7.4, 140 mM NaCl, 5 mM KCl, 1 mM MgCl2, 1 mM CaCl2, 24 mM D-Glucose), and cut into 500 μm coronal sections using a vibroslice vibratome (752M, Camden Instruments, Leica, UK). The resulting SCN slices were cultured in oxygenated ACSF for at least 60 minutes at 35°C. After incubation, slices were bisected along the third ventricle, separating the 'left' and 'right' SCN, and transferred into a new media containing the treatment (20 nM EGF or vehicle). This concentration was selected because previous work in hepatocytes [50] showed that saturating levels of EGFR activation are achieved with 20 nM levels of EGF; similarly, studies using lower concentrations in hippocampal brain slices have observed robust responses [51,52]. Slices were incubated in treatment for 60 minutes before taking 0.75 mm micropunches (Stoelting, Chicago, IL, USA). Punches for day treatment were taken at 2 PM (8 hours after lights on) while punches for night treatment were taken at 8 PM (2 hours after lights off). RNA was extracted using the Rneasy mini kit (Qiagen, Valencia, CA, USA), yielding approximately 200 nanograms of total RNA/punch. Two hundred to four hundred nanograms total RNA were amplified using two rounds of antisense RNA (aRNA) amplification using the RNA MessageAmp kit (Ambion, Austin, TX, USA), yielding no less than 130 μg aRNA. aRNA quality was assessed using Bioanalyzer Picochip (Agilent Technologies, Palo Alto, CA, USA), and 2.25 μg of aRNA was used to generate amino-allyl and Cy dye conjugated labeled cDNA (Cy5, Amersham Pharmacia Biotech, Piscataway, NJ, USA) using the indirect aminoallyl-dNTP approach. Experimental details for microarray hybridization, scanning, and quantification are in Additional data file 5.

### Microarrays

In-house constructed cDNA microarrays containing 2,700 unique University of Iowa Rat clones from all rat tissues spotted at least twice per slide (5,464 spots split across 48 subarrays per slide) onto 1' by 3' glass microarray slides (Corning, Corning, NY, USA) were used in the present study. Clones were selected on the basis of possessing good quality GO annotation and available promoter sequences. PCR amplicons were prepared from freshly grown overnight cultures of clones for printing using GF200 primers. Following amplification and purification, amplicons were resuspended in 20 μL of 50% DMSO and printed onto UltraGAPs aminosilane-coated slides (Corning) using a MicroGridTAS (Genomic Solutions, Ann Arbor, MI, USA) rearrayer. After printing, DNA was cross-linked to the slides by UV irradiation with a Stratalinker UV Crosslinker (Stratagene, La Jolla, CA, USA) and stored in a vacuum chamber until use.

### Microarray data normalization

Normalization of microarray data was performed using a random effects ANOVA model with terms for slide, subarray, and slide:subarray interactions, similar to [53,54]. Following [54], additional normalization was accomplished by scaling the ANOVA model residuals with the subarray specific standard deviations to standardize the dispersion. This two-step normalization procedure is referred to as 'standard normalization' throughout the manuscript. Computations were performed using the package NLME [20] in the statistical analysis environment R [55].

### Identification and classification of EGF-responsive genes

**Figure 5:**
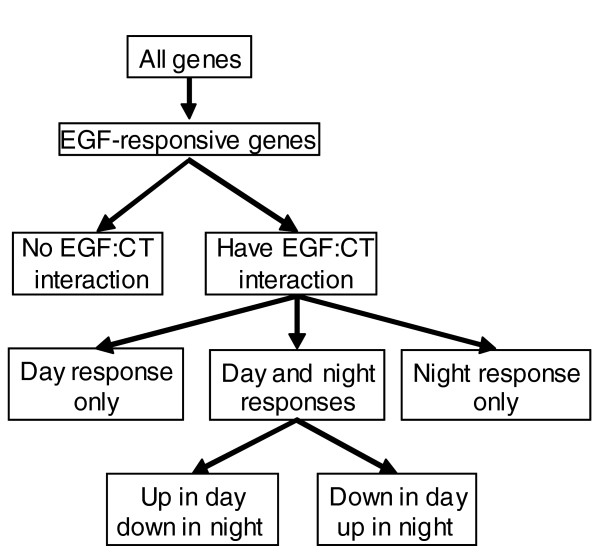
Hierarchical analysis approach. Genes were first classified as EGF responsive by performing a likelihood ratio test to compare the fits of maximum likelihood estimated mixed models with and without EGF terms. EGF responsive genes were then classified as to whether or not they had a significant EGF:CT interaction, as determined by a Wald F-test of the REML estimated full model. EGF responsive genes with EGF:CT interactions were then classified according to whether they were responsive to EGF during the day only, during the night only, or at both times. Genes with significant EGF:CT interactions were then subdivided according to the directionality ofthe responses.

Genes with EGF responses modulated by circadian time were identified using mixed-model ANOVA. Following previous studies [53,56], mixed models with terms for the fixed effects of interest and obscuring random effects were fit to normalized expression data for each gene:

log_2_(*y*_*ijkl*_) = μ + *E*_*i *_+ *C*_*j *_+ *EC*_*ij *_+ *R*_*k *_+ ε_*ijl*(*k*) _    (1)

where: *y*_*ijkl *_is the normalized expression level of a specific gene; *E *= EGF treatment fixed effect (*i *= 0 for vehicle; *i *= 1 for EGF); *C *= circadian time fixed effect (*j *= 0 for Day; *j *= 1 for Night); *R *= *N*(0, σ_R_^2^) rat random effect (*k *= (*a*, *b*, *c*, *d*) for the four rats used); and ε_*ijl*(*k*) _= *N*(0, σ_ε_^2^) residual error (where *N*(0, *v*) indicates a normal distribution with zero mean and variance *v*). Indices for spots of genes repeated on a single array are given by subscript *l *(*l *= 1 for *N*_*s*_, where *N*_*s *_= number of spots per array for that gene; *N*_*s *_= 2 for most genes). Models were fit using maximum likelihood and restricted maximum likelihood (REML) algorithms in the R package NLME [20]. Genes with EGF responses, circadian time dependent EGF responses, and specific EGF:CT interactions were identified by fitting Equation 1 and reduced versions with terms removed. The overall hierarchical classification procedure is shown in Figure 5 and details are given in Additional data file 5.

### Enrichment analyses

Hypotheses for SCN circadian time dependent EGF signaling regulation were generated by testing EGF-responsive gene groups for enrichment of functional attributes. Evidence for modulation of core circadian clock function by EGFR signaling was obtained by testing for enrichments of established SCN circadian genes [17,22] in the EGF responsive gene groups. Cellular process/function hypotheses were generated from GO term enrichments. Transcriptional regulatory hypotheses were based on enrichments of predicted TF binding activities, referred to as transcriptional regulatory network analysis. Three sources of TF binding predictions were used: TRANSFAC Pro database of TF binding sites with the MATCH tool [26] as automated using PAINT [21]; predictions based on phylogenetic conservation of TF binding sites from [27]; and protein-DNA interaction data for CREB [28] and three HNF family members [29]. Genes too long to show differential transcription induced expression changes after one hour of EGF treatment were excluded from the transcriptional regulatory network analysis (genes >75,000 base pairs (bp), assuming 1,500 bp/minute elongation [57] and 10 minute processing [58]). Fisher's exact test was used to compute enrichment *p *values (*p*_ENRICH_), using all microarray genes as the reference, following [21]. Gene attributes (GO or TF binding) not present in at least five genes in a particular gene group were not tested. Fisher's test p values were FDR adjusted based on [59]. Further enrichment analysis details are in Additional data file 5.

### Meta-analysis of enrichments

Enrichment of GO or TF binding sites in different gene groups can depend nonlinearly on the parameters used to define significantly differentially expressed gene groups [25]. Furthermore, microarray results can depend somewhat on the normalization approach employed [54]. Ideally, enrichment results will reflect waves of regulation and, therefore, will be robust to small variations in significance cut offs and normalization techniques. To identify enrichment results insensitive to these variables, we supplemented the gene group analyses by computing two additional *p *values, *p*_M_^(LOCAL) ^and *p*_M_^(GLOBAL)^, which are local and global meta-analysis *p *values, respectively. *p*_M_^(LOCAL) ^is the geometric mean (GM) of the enrichment *p *values (*p*_ENRICH_) computed for gene groups defined using three cut offs for significance for the standard normalization. *p*_M_^(GLOBAL) ^is the GM of the enrichment *p *values computed for gene groups defined using the three significance cut offs and 8 slightly different normalization techniques, a total of 24 conditions. Attributes with low values of *p*_M_^(LOCAL) ^and *p*_M_^(GLOBAL) ^are 'robustly enriched' because their overall enrichment is not dependent on a particular significance cut off or normalization. The cut off for 'locally robust enrichment' was *p*_M_^(LOCAL) ^< 0.06 while it was *p*_M_^(GLOBAL) ^< 0.10 for 'globally robust enrichment'. Full details are in Additional data file 5.

### qRT-PCR testing of implicated TFs

Expression levels of TFs implicated in circadian time dependent responses to EGF in the SCN (*c-Jun*, *c-Ets1*, *Creb1*, *C/EBPα, C/EBPβ, C/EBPγ, Rorα *and *Rorβ*) were measured using qRT-PCR. *Fyn *was used as a housekeeping control gene given that two independent *Fyn *clones had no significant EGF or circadian time effects and relatively small experimental variability on our microarrays. aRNA from the microarray samples and two additional daytime samples were used for qRT-PCR.

The analysis approach used for the qRT-PCR data was a combination of the 'ΔΔ*Ct*' method [60] and the mixed-model ANOVA employed for the microarray analysis. The approximate range of exponential growth for each gene in each well was first determined from the amplification curves using a procedure modified from [61].

Studentized residuals were used to detect the onset of the exponential growth phase from the background subtracted (DeltaRn) reaction curve. The beginning of exponential growth was defined as the point after which four outlier points were detected in a row at *p <*0.025. The termination of the exponential growth phase was defined as the point at which the slope of the log-transformed amplification curve (as estimated using linear regression over a window of five cycles) first differs from the initial slope of the exponential phase (as estimated using linear regression over a window of five cycles) with 95% confidence. Regressions and computations were performed using the *stats *package in the statistical analysis environment R [55]. PCR reactions that did not amplify (did not reach a raw intensity of 0.2) were excluded from the analysis.

With regions of exponential growth defined for each gene in each condition, it was possible to compute delta-cycle-thresholds (Δ*Cyt*_*g*_) for each gene (*g*) with respect to the housekeeping gene (*h*), such that:

Δ*Cyt*_*g *_(*I*) = *Cyt*_*g*_(*I*) *- Cyt*_*h*_(*I*)     (2)

where *I *is the 'threshold intensity' - the intensity of the amplification curve at which the cycle thresholds (*Cyt*) for each gene and the housekeeping gene are estimated. If both the housekeeping gene and the gene of interest are in exponential phases with the same efficiency, the Δ*Cyt*_*g *_should not depend on *I *and will be proportional to *-log*2 the relative expression level (*-log*2([*g*]*/ *[*h*])) if the efficiencies are perfect (= 2), where [*g*] is the transcript concentration of the gene of interest and [*h*] is the transcript concentration of the housekeeping gene. Δ*Cyt*_*g*_, however, generally shows some weak nonlinear dependence on *I*. Estimation of the exponential phases allows identification of the intensity regions where amplicons for both the housekeeping gene and the gene of interest are undergoing exponential growth. If that overlap spans several PCR cycles for both genes, multiple estimates of Δ*Cyt*_*g *_can be obtained by interpolation of the amplification curves. This was true in all cases presently. For each gene of interest in each condition, the amplification curves were interpolated on an exponential scale in the region of overlap with the housekeeping gene to obtain *n *+ 1 estimates of Δ*Cyt*_*g*_, where *n *is the minimum number PCR cycles (between the gene of interest and the housekeeping gene) where overlap of the exponential phases occurs. The multiple estimates of Δ*Cyt*_*g*_(*I*) were used to compute an overall average Δ*Cyt*_*g*, *av *_that was used in the subsequent analyses.

Δ*Cyt*_*g*, *av *_was computed as described above for each gene for each condition on each PCR plate used in the measurements. To account for any plate or plate-region specific bias, the mean Δ*Cyt*_*g*, *av *_across all conditions for gene (*g*) on the same plate or region was subtracted, to give normalized (Δ*Cyt*_*g*, *norm*_) values for analysis. The qRT-PCR data retain the factorial mixed-effects structure of the microarray data, and for this reason mixed model ANOVA was again employed to identify statistically significant effects on gene expression. Specifically, the following model was used (where the subscripts on Δ*Cyt*_*g*, *norm *_have been removed for clarity):

-Δ*Cyt*_*ijklm *_= *μ *+ *E*_*i *_+ *C*_*j *_+ *EC*_*ij *_+ *R*_*k *_+ ε_*ijkm *_    (3)

The terms on the right-hand side of Equation 3 have the same meaning as the terms in Equation 1, except that *l*, the index associated with spots (nested within rats), has been replaced with the index *m*, an index associated with PCR plates or PCR plate quadrants in which measurements were taken. Since measurements of all conditions were obtained on each PCR plate, PCR plates are not nested in rats. The parameters in Equation 3 were estimated for each gene using REML. Wald *F *tests were then performed to determine whether any of the fixed effects were statistically significant. As above, computations were performed using the NLME package in R [20].

Lastly, we undertook an analysis to determine whether the 'overall' correlations between the TF expression profiles and the EGF responsive gene groups were greater than the correlations between the TFs and random gene groups of the same size. Details of this analysis are provided in Additional data file 5.

### Data deposition

Raw expression data for the present study has been submitted to the NCBI Gene Expression Omnibus as series GSE4245.

## Additional data files

The following additional data are available with the online version of this paper. Additional data file 1 is a figure showing gene expression boxplots for several genes with specific circadian time dependent EGF responses in the SCN. Additional data file 2 is a figure showing gene expression boxplots for the transcription factors investigated by qRT-PCR. Additional data file 3 is a table listing *p *values for EGF effects and EGF:CT interactions for all genes present on the microarrays of the current study. Additional data file 4 is a table listing *p *values for EGF effects and EGF:CT interactions for SCN circadian genes [17] present on the microarrays of the current study. Additional data file 5 provides more detailed materials and methods. Additional data file 6 provides supplementary results.

## Supplementary Material

Additional data file 1 Gene expression boxplots for several genes with specific circadian time dependent EGF responses in the SCN. Click here for file

Additional data file 2 Gene expression boxplots for the transcription factors investigated by qRT-PCR. Click here for file

Additional data file 3 P values for EGF effects and EGF:CT interactions for all genes present on the microarrays of the current study. Click here for file

Additional data file 4 P values for EGF effects and EGF:CT interactions for SCN circadian genes [17] present on the microarrays of the current study. Click here for file

Additional data file 5 Detailed materials and methods. Click here for file

Additional data file 6 Additional results. Click here for file

## Figures and Tables

**Table 1:** EGF responsive genes in the SCN are involved in diverse cellular processes and potentially regulated by diverse transcription factors

**Gene group**	**Attribute class**	**Attribute**	***p***_ENRICH_	***p***_ENRICH_^(FDR)^	**No. of genes**	**G**_FRAC_	**A**_FRAC_	**ENRICH**	***p***_M_^(LOCAL)^	***p***_M_^(GLOBAL)^
**Any EGF effect**	GO	**Cell differentiation**	**3.E-03**	**0.08**	**7**	**0.1**	**0.3**	**3.4**	**0.02**	**0.06**
		**Protein kinase activity**	**0.01**	**0.10**	**10**	**0.2**	**0.2**	**2.4**	**0.03**	**0.08**
		**Protein serine/threonine kinase activity**	**2.E-03**	**0.08**	**10**	**0.2**	**0.2**	**2.8**	**0.01**	**0.06**
	PAINT	ATF3	0.02	0.16	5	0.1	0.3	2.9	0.02	0.52
		**CREB**	**4.E-03**	**0.06**	**9**	**0.1**	**0.3**	**2.7**	**4.E-03**	**0.10**
		CREBATF	3.E-03	0.06	5	0.1	0.5	4.3	0.04	0.59
		CRE-BP1:c-Jun	0.01	0.14	7	0.1	0.3	2.6	0.02	0.31
	CONS	V$AP1_2	0.02	0.90	71	0.8	0.1	1.1	0.03	0.11
		**V$AP1_C**	**1.E-03**	**0.16**	**53**	**0.6**	**0.2**	**1.4**	**2.E-03**	**0.06**
		**V$CEBP_Q2**	**0.01**	**0.54**	**32**	**0.4**	**0.2**	**1.4**	**0.01**	**0.03**
		**V$CEBP_Q2_01**	**0.03**	**0.90**	**61**	**0.7**	**0.1**	**1.2**	**0.05**	**0.06**
		**V$CEBP_Q3**	**2.E-03**	**0.19**	**69**	**0.8**	**0.1**	**1.2**	**2.E-03**	**0.02**
		**V$CEBPGAMMA_Q6**	**0.03**	**0.90**	**26**	**0.3**	**0.2**	**1.4**	**0.05**	**0.07**
		V$OCT1_07	0.02	0.90	10	0.1	0.2	2.0	0.05	0.23
		**V$RORA1_01**	**7.E-05**	**0.02**	**21**	**0.2**	**0.3**	**2.3**	**3.E-04**	**2.E-03**
		V$RORA2_01	0.01	0.54	6	0.1	0.4	3.1	0.06	0.19
	Circ.	**SCN circadian genes [17]**	**0.04**	**-**	**16**	**0.1**	**0.4**	**1.5**	**0.02**	**-**
										
**EGF:CT**	GO	**Cell differentiation**	**1.E-03**	**0.06**	**6**	**0.1**	**0.2**	**4.5**	**0.01**	**0.05**
**interaction**	PAINT	c-Ets-1/68	0.07	0.36	8	0.1	0.2	1.8	0.06	0.13
		CREB	0.01	0.10	7	0.1	0.2	2.7	0.01	0.11
		CREBATF	8.E-04	0.02	5	0.1	0.5	5.7	0.02	0.60
		CRE-BP1:c-Jun	0.01	0.10	6	0.1	0.3	3.0	0.01	0.23
		E2F	0.03	0.20	5	0.1	0.2	2.7	0.02	0.28
	CONS	**V$AP1_C**	**2.E-04**	**0.03**	**41**	**0.7**	**0.1**	**1.5**	**2.E-03**	**0.05**
		**V$CEBP_Q2**	**2.E-03**	**0.11**	**26**	**0.4**	**0.1**	**1.7**	**0.01**	**0.07**
		**V$CEBP_Q3**	**5.E-03**	**0.26**	**49**	**0.8**	**0.1**	**1.2**	**0.01**	**0.09**
		V$CETS1P54_01	0.03	0.71	46	0.7	0.1	1.2	0.05	0.13
		V$CREBP1_Q2	0.03	0.71	8	0.1	0.2	2.1	0.04	0.36
		V$ER_Q6_02	0.02	0.69	30	0.5	0.1	1.4	0.01	0.10
		**V$HFH4_01**	**0.01**	**0.31**	**8**	**0.1**	**0.2**	**2.7**	**0.01**	**0.08**
		**V$RORA1_01**	**2.E-04**	**0.03**	**16**	**0.3**	**0.2**	**2.5**	**1.E-03**	**4.E-03**
		V$RORA2_01	1.E-03	0.11	6	0.1	0.4	4.4	0.03	0.14
	ChIP	HNF1-alpha	0.04	0.11	6	0.1	0.2	2.3	0.15	0.25
	Circ.	SCN circadian genes [17]	0.05	-	13	0.1	0.3	1.6	0.07	-
										
**EGF without interaction**	GO	**Protein binding**	**0.02**	**0.06**	**7**	**0.3**	**0.1**	**2.5**	**0.01**	**0.06**
		Protein kinase activity	2.E-03	0.02	6	0.3	0.1	4.2	0.03	0.37
		Protein serine/threonine kinase activity	1.E-03	0.02	6	0.3	0.1	4.8	0.02	0.32
		Transferase activity	2.E-03	0.02	9	0.4	0.1	2.8	0.04	0.22
	CONS	**V$CEBPGAMMA_Q6**	**0.02**	**1**	**11**	**0.4**	**0.1**	**1.9**	**0.01**	**0.08**
		**V$TFIIA_Q6**	**0.02**	**1**	**12**	**0.4**	**0.1**	**1.8**	**0.03**	**0.04**
										
**EGF: day+night**	CONS	V$CREB_Q2	2.E-03	0.15	5	0.4	0.1	4.8	0.01	0.65
		V$CREB_Q4	9.E-04	0.12	6	0.5	0.1	4.5	4.E-03	0.55
		V$CREB_Q4_01	4.E-03	0.19	7	0.5	0.05	2.9	0.01	0.50
	ChIP	CREB (relaxed)	0.11	0.11	6	0.5	0.03	1.7	0.24	0.35
										
**EGF: night only**	CONS	V$AP1_C	3.E-03	0.14	27	0.7	0.1	1.5	0.03	0.17
		**V$CEBP_Q2**	**6.E-04**	**0.13**	**20**	**0.5**	**0.1**	**2.0**	**0.01**	**0.08**
		V$CEBP_Q2_01	0.02	0.40	31	0.8	0.1	1.3	0.04	0.16
		**V$CEBP_Q3**	**5.E-03**	**0.16**	**34**	**0.8**	**0.1**	**1.3**	**0.01**	**0.09**
		**V$CREBP1_01**	**0.01**	**0.31**	**8**	**0.2**	**0.1**	**2.5**	**0.03**	**0.04**
		V$ER_Q6_02	4.E-03	0.16	23	0.6	0.1	1.6	0.02	0.15
		V$HFH4_01	2.E-03	0.13	7	0.2	0.2	3.6	4.E-03	0.20
		V$LMO2COM_02	0.02	0.43	29	0.7	0.1	1.3	0.06	0.13
		**V$RORA1_01**	**2.E-03**	**0.13**	**11**	**0.3**	**0.1**	**2.6**	**0.01**	**0.01**
		**V$RORA2_01**	**1.E-03**	**0.13**	**5**	**0.1**	**0.3**	**5.5**	**0.02**	**0.08**

Statistically significant enrichments for specific cellular functions or TF binding sites (Attribute) are given for gene groups with specific circadian time dependent EGF responses (Gene group). Distinct gene groups are enriched for distinct and overlapping functions and TF binding sites. GO, gene ontology functional annotation; PAINT, TF binding sites predictions using PAINT [21]; CONS, TF binding sites based on evolutionary conservation [27]; ChIP, TF binding predictions based on the protein-DNA interaction data [28, 29]; Circ., established circadian rhythmic SCN gene expression [17]; p_ENRICH_, gene group enrichment *p *value; *p*_ENRICH_^(FDR)^, false discovery rate (FDR) adjusted *p*_ENRICH_; No. of genes, number of genes in gene group with the attribute; G_FRAC_, fraction of genes in the gene group with the attribute; A_FRAC_, fraction of all genes on the microarray with the attribute that are in the gene group; ENRICH, fold enrichment of the attribute in the gene group over random; *p*_M_^(LOCAL)^, local meta-analysis enrichment p value; *p*_M_^(GLOBAL)^, global meta-analysis enrichment *p *value. *p*_ENRICH_, *p*_ENRICH_^(FDR)^, No. of genes, G_FRAC_, A_FRAC_, and ENRICH values are for results obtained using the standard normalization and are based on gene groups defined at a significance threshold of 1% FDR for GO enrichments, a significance threshold of 2% for PAINT, CONS and ChIP enrichments, and a significance threshold of 5% for circadian gene enrichments. *p*_M_^(LOCAL) ^values are for standard normalization results and gene group significance thresholds of 5%, 2%, and 1% FDR for GO, PAINT, CONS, and ChIP enrichments and gene group significance thresholds of 20%, 10%, and 5% FDR for Circ. enrichments. Emphasized attributes are robust as indicated by both meta-analysis *p *values (*p*_M_^(LOCAL) ^< 0.06 and *p*_M_^(GLOBAL) ^< 0.1).
