# Supplementary material for: Systems analysis of circadian time-dependent neuronal epidermal growth factor receptor signaling
Source: Genome Biol. 2006 Jun 19;7(6):R48. doi: 10.1186/gb-2006-7-6-r48 (PMC1779538; doi:10.1186/gb-2006-7-6-r48)
Supplement: Additional data file 1 — Gene expression boxplots for several genes with specific circadian time dependent EGF responses in the SCN. [file gb-2006-7-6-r48-S1.doc]

**Supporting figure 1: Select expression responses to EGFR activation in the SCN.** “C” = control during the day, “CN” = control during the night, “E” = EGF treatment during the day, and “EN” = EGF treatment during the night. Boxes indicate maximum, mean, and minimum normalized expression values. Night responses are shaded.
