# Supplementary material for: Systems analysis of circadian time-dependent neuronal epidermal growth factor receptor signaling
Source: Genome Biol. 2006 Jun 19;7(6):R48. doi: 10.1186/gb-2006-7-6-r48 (PMC1779538; doi:10.1186/gb-2006-7-6-r48)
Supplement: Additional data file 2 — Gene expression boxplots for the transcription factors investigated by qRT-PCR. [file gb-2006-7-6-r48-S2.doc]

**Supporting Figure 2: Transcriptional responses of selected TFs to EGFR activation in the SCN as measured by qRT-PCR.** Shown are normalized -ΔCt values (~log_2_ normalized expression levels – see Materials & Methods) for different experimental conditions for *c-Jun*, *c-Ets1*, *Creb1*, *c/EBPα,* *c/EBPβ*, and *c/EBPγ*. “C” = control during the day, “CN” = control during the night, “E” = EGF treatment during the day, and “EN” = EGF treatment during the night. Night responses are shaded. *C-Jun* and *C/EBPγ* are consistently down-regulated during both day and night, *c-Ets1* and *Creb1* are both down-regulated during the day and up-regulated during the night, *c/EBPα* is consistently up-regulated during the night, and *c/EBPβ* is consistently down-regulated during the day only. *C/EBPγ* transcripts were not detected in “*Rat e*” samples.
