# Supplementary material for: Systems analysis of circadian time-dependent neuronal epidermal growth factor receptor signaling
Source: Genome Biol. 2006 Jun 19;7(6):R48. doi: 10.1186/gb-2006-7-6-r48 (PMC1779538; doi:10.1186/gb-2006-7-6-r48)
Supplement: Additional data file 5 — Detailed materials and methods. [file gb-2006-7-6-r48-S5.doc]

**Supporting information: Detailed Materials & Methods**

***Hybridization, scanning, and quantification***

Microarray slides were prehybridized in 10mg/mL bovine serum albumin, 5X SSC, 0.1% SDS for 45 minutes at 42°C, after which the slides were rinsed with water and dried. Cy3- and Cy5-labeled cDNA was resuspended in 50 µl of DIG Easy Hyb solution (Roche Applied Science, Indianapolis, IN) with yeast tRNA (0.5 mg/ml) and calf thymus DNA (0.5 mg/ml) and hybridized to the microarray at 37°C for 18 hours under glass coverslips. Following hybridization, slides were washed with agitation for 15 minutes at 50°C in solution containing 1X SSC and 1% SDS, followed by an 1-minute wash of 1X SSC at room temperature; then by three 1-minute washes of 0.1X SSC at room temperature. Slides were dried by centrifugation.

After drying, slides were scanned at 10 µm resolution using a PerkinElmer scanner, and were quantified using the ScanArray Express v2.2 program (Perkin Elmer, Wellesley, MA), with Adaptive Threshold segmentation. Clones that lacked detectable spotted cDNA (no detectable signal on the Cy3 vector reference channel), were saturated (at least one spot with an intensity *>* 65535) on the Cy3 or Cy5 channels, or were in the background (Cy5 or Cy3 intensities that were within the 95% for the background of the respective channel), were excluded, leaving 1966 clones, out of the original 2,700 clones spotted on the array, for subsequent analysis.

***Hierarchical microarray analysis procedure***

The objective of the analysis was to identify genes with expression responses to EGFR activation in the SCN, and then to identify subsets of those genes with responses that were modulated by circadian time (CT). Following other studies [53, 74, 56], mixed-model ANOVA was employed to achieve these analysis objectives. The following two models were first fit to data for each gene:

$\log_{2}(y_{ijkl})=\mu+E_{i}+C_{j}+(EC)_{ij}+R_{k}+\varepsilon_{ijl(k)}$ Equation S1

$\log_{2}(y_{ijkl})=\mu+C_{j}+R_{k}+\varepsilon_{ijl(k)}$ Equation S2

where *y* is the normalized expression level of a specific gene; *E*=EGF treatment fixed effect (*i* = 0: vehicle; *i* = 1: EGF); *C* = circadian time (CT) fixed effect (*j* = 0: Day, *j* = 1: Night); *R* = *N*(0, σ_R_^2^) rat random effect (*k* = (*a*, *b*, *c*, *d*) for the four rats used); and ε*_ijl(k)_* = *N*(0, σ_ε_^2^) residual error. Indices for spots of genes repeated on a single array are given by subscript *l* (*l* = 1: *N_s_*, where *N_s_* = number of spots/array for that gene. *N_s_* = 2 for most genes). In Equations S1 and S2, array-array variation is lumped together with spot-spot variation in the residual term ε, in a similar manner as [56]. The mixed-models were fit using maximum likelihood (ML) and restricted maximum likelihood (REML) as implemented in the NLME package for R [20]. As a preliminary filtering step, genes with estimates of $\sigma_{R}$ that had very broad 95% confidence intervals ($\sigma_{R}^{upper}$*>* 10, keeping in mind that $\sigma_{R}$ is on a *log*2 scale) were excluded from subsequent analysis. This is because the models for those genes were essentially unidentifiable from the data given high levels of noise (*practically* unidentifiable,[75]) [20]. Confidence intervals were computed using the function *confint* in the NLME package.

Approximately thirteen hundred genes remained for subsequent analyses. EGF-responsive genes were defined as those for which the fit to Equation S1 (the model with EGF terms) was statistically significantly improved over the fit to Equation S2 (the model without EGF terms), an approach similar to that used by [19] for identifying estrogen responsive genes in cancer cells. This approach was used instead of simply testing for significant *E* or *EC* effects in Equation S1 because genes with *any significant EGF effect at all* are identified with a single test, simplifying the subsequent analysis somewhat. It also would be valid to inspect the p-values for *E* and *EC* effects in parallel to identify genes that were significantly affected by EGF. The significance of the improvement in fit was assessed by a likelihood ratio test of the maximum likelihood estimates of the models in Equation S1 and Equation S2 for each gene, giving a p-value for being affected by EGF in some way, *p^EGF^*. The issue of multiple testing that arises from performing ~2000 likelihood ratio tests for ~2000 genes was addressed by computing false discovery rate (FDR, [59]) adjusted p-values. A nominal significance cutoff of *p^EGF^ <* 0.02 was used for the FDR adjusted p-values. Likelihood ratio tests were carried out using the NLME package in R [20], and p-value adjustments were carried out using the R package Multtest.

Once EGF-responsive genes were identified, it was of interest to identify the subset of those genes that had EGF responses modulated by circadian time. This was accomplished using Wald F-tests for statistical significance on the EGF:CT interaction term ((*EC*)*_ij_*) in the REML estimates of Equation S1, giving a p-value for EGF:CT interactions, *p^EGF:CT^*. Multiple testing is less of an issue for these interaction tests than it was for the tests of EGF responses because interaction tests were only performed on the EGF responsive gene subset. Nevertheless, multiple testing was accounted for using FDR adjustment of the p-values. The Wald F-tests were carried out using the NLME package in R [20].

EGF responsive genes with EGF:CT interactions can be responsive to EGF during one of the circadian times only (day or night), or they can be responsive to EGF at both but with differing signs or magnitudes of responses. These possibilities can be differentiated by testing different contrasts of the EGF, CT, and EGF:CT effects [76]. A simpler approach is employed presently, based on the following rationale: EGF responsive genes with EGF:CT interactions should be differentially expressed in response to EGF for at least one circadian time. Two additional mixed models were thus fit for each EGF responsive gene with a significant EGF:CT interaction: one for each circadian time (half of the data), shown below.

$\log_{2}(y_{i0kl})=\mu+E_{i}+R_{k}+\varepsilon_{i0l(k)}$ Equation S3

$\log_{2}(y_{i1kl})=\mu+E_{i}+R_{k}+\varepsilon_{i1l(k)}$ Equation S4

Equation S3 corresponds to the daytime EGF response (*j* = 0) and Equation S4 corresponds to the nighttime EGF response (*j* = 1). Wald F-tests were performed to obtain p-values for the daytime and nighttime EGF effects for each gene *g*, $p_{g}^{EGF,Day}$ and$p_{g}^{EGF,Night}$, respectively. The minimum p-value for each gene for day and night EGF effects ($p_{g}^{MIN}=\min(p_{g}^{EGF,Day},p_{g}^{EGF,Night})$) was computed. The maximum value of $p_{g}^{MIN}$ over all genes ($p_{ALL}^{MAX}$ = *max*($p_{g}^{MIN}$)) was then designated as a cutoff for significant EGF effects (ensuring that each gene would have a significant EGF effect during at least one circadian time: ($p_{g}^{MIN}\leq p_{ALL}^{MAX} \forall g$). Genes for which $p_{g}^{EGF,Day}\leq p_{ALL}^{MAX}$**and** $p_{g}^{EGF,Night}\geq2\times p_{ALL}^{MAX}$, were designated as EGF responsive during the day only. The interpretation of these conditions is that the daytime EGF effect was significant while the nighttime EGF effect was not, and that the null hypothesis of no daytime EGF effect was at least twice as likely to be rejected as the null hypothesis for no nighttime EGF effect. The second condition was added to avoid somewhat arbitrary designations that would occur, for example, if $p_{ALL}^{MAX}$ = 0*.*05, $p_{g}^{EGF,Day}$= 0*.*045, and $p_{g}^{EGF,Night}$= 0.055: the day effect would be declared significant and the night effect insignificant, even though the null hypothesis for a daytime effect is only 1.2 times as likely to be rejected as the null hypothesis for a nighttime effect. Similar conditions were used to define genes that were responsive to EGF during the circadian night only. Genes that did not meet the conditions for being specifically day or night responsive were designated as being responsive at both times.

EGF responsive genes with significant EGF:CT interactions and EGF responses at both circadian times were subdivided based on the directionality of the responses to EGF (as determined by the signs of the effects from Equation S3 or Equation S4). There are four possibilities: (1) the genes can be significantly up-regulated by EGF during both day and night, with the magnitude of the up-regulation differing significantly; (2) the genes can be down-regulated by EGF at both times, with the magnitude of the changes being circadian time dependent; (3) the genes can be up-regulated during the day by EGF and down-regulated by EGF at night; or (4) the genes can be down-regulated during the day and up-regulated during the night. Interestingly, appreciable numbers of genes were found only for the latter two cases (EGF responses with opposing directionality). The overall approach to identifying EGF responsive genes with specific EGF:CT interactions, described above, is shown in Figure 5 of the main text.

***Gene group enrichment analyses***

As described in the main text, hypotheses for regulation of circadian time dependent EGF responses were generated by testing EGF-responsive gene groups for enrichment of functional attributes. These attributes included prior designation as genes with circadian patterns of expression [17, 22], Gene Ontology (GO) terms, and three types of transcription factor (TF) binding predictions. Details about the tests for statistically significant enrichment and each specific functional gene attribute are given below.

*Testing for statistically significant gene group enrichments*

Statistically significant gene group enrichments were tested for using Fisher’s exact test. This statistical test, based on the hypergeometric probability distribution, gives the exact probability of obtaining at least *x* red balls when drawing *m* balls from an urn containing *N* balls of which *Y* are red. In the context of gene group enrichments, the reference “urn” is the set of all genes that were tested for EGF responses (not saturated, not background, without very broad σ_R_ confidence intervals), the “red balls” are genes with a particular functional attribute (ex: associated with a particular GO term or having a particular TF binding site in their promoters), and the “drawing” process is the classification of groups of genes according to specific EGF responses/interactions with the circadian time. This test has been used extensively in functional genomics studies [77, 78, 21]. The multiple testing problem that arises from testing for enrichment of 100s of functional attributes of a certain class was accounted for by computing FDR adjusted p-values. Only attributes that annotated at least five genes in a particular gene group, however, were tested for enrichment. This is because the functional properties of ensembles of genes are of interest, not the chance association of one or two genes with rare attributes. It follows that the number of enrichment tests performed, and therefore the multiple testing correction, depends on the size and character of the gene groups themselves. For example, in testing for GO enrichment of a gene group consisting of five genes that all shared a single GO term, only one test would be performed, eliminating the need for multiple testing corrections for that gene group. The enrichment tests for TF binding predictions were identical to the tests for enrichment of GO terms and circadian expression, with the exception that very long genes were excluded entirely from the TF enrichment analysis (removed from the “urn”). In particular, genes with genomic lengths > 75,000 bp were excluded. The reason is that these genes require at least 60 minutes to be transcribed and processed, assuming 1500 bp/min elongation [57] and 10 min processing [58]. Our expression measurements were made 60 minutes after EGF treatment, and thus any differential gene expression changes observed in these genes could not be due to transcriptional regulation. Since testing for gene group enrichment of TF binding site predictions yields hypotheses about transcriptional regulation exclusively, these candidate post-transcriptional regulated genes will only contribute noise to the analysis. Gene lengths were determined using the UCSC Genome Browser Database [79].

*Circadian gene expression attributes*

In order to determine if the EGF-responsive gene targets we identified were relevant to circadian clock function, we compared them to previously established circadian cycling genes in the SCN [17, 22]. Specifically, we tested whether circadian-regulated genes were over-represented in our EGF-responsive gene groups compared to random gene groups of the same size. To perform the test, we first obtained updated (12/20/2005) annotation files for the microarrays used in the previous studies (Affymetrix MG_U74a and MG_U74av2 Gene Chip arrays for [17] and [22], respectively) from the Affymetrix web site (http://www.affymetrix.com/). We then mapped the mouse genes from those arrays to their rat homologues on our arrays using Homologene [37], giving reference sets of 757 genes and 772 genes, respectively for the datasets in [17] and [22], respectively. Of the genes identified to have circadian variations in expression levels, 38 out of the 365 and 15 out of 101 genes reported in [17] and [22], respectively, were represented in clones on our arrays. Given this relatively low representation of circadian genes on our arrays, we performed the enrichment analysis using less stringent significance cutoffs than for the other attributes. Specifically, we set the primary gene groups significance cutoff as FDR < 10% for the base case.

*GO functional attributes*

The genes on our microarrays were associated with GO attributes by using a tool that first related clone identifiers to Locuslink identifiers through UniGene, and then related Locuslink identifiers to gene ontology (GO) terms using Entrez Gene. The parents of the GO terms were found using the GO database (http://www.godatabase.org) and annotated as containing the superset of all genes contained in their children. About 63% of the genes on the arrays were annotated with at least one GO term, and 719 GO terms annotate at least five genes on the arrays. GO terms that were associated with less than five genes on our arrays were excluded from the analysis. GO terms can be highly correlated with one another in the sets of genes that they annotate. To address this issue, GO terms that were highly correlated, as measured by Jaccard similarity coefficients, were agglomerated. The Jaccard similarity between any two GO terms is the number of genes annotated with both terms divided by the number of genes annotated with at least one of the terms. Examples of highly correlated GO terms are “phosphate metabolism” and “phosphorus metabolism.” GO terms with 90% Jaccard similarity or greater were agglomerated, leaving 551 GO terms for gene group enrichment tests. Computation of Jaccard similarities between GO terms was accomplished using the Prabclus package in R.

*TF binding attributes: predictions using PAINT*

The objective of transcriptional regulatory network analysis - testing groups of similarly expressed genes for enrichment of specific TF binding site predictions in their promoters - is to make hypotheses about the TFs actively regulating particular expression responses. As evinced by ~600 citations of the pioneering work in [77] (http://scholar.google.com), and the numerous examples in mammalian systems (for example, [21, 80, 81, 24], there is widespread interest in transcriptional regulatory network analysis. The Promoter Analysis and Interaction Network Toolset (PAINT, [21]) automates transcriptional regulatory network analysis: for any group of genes, PAINT retrieves the cognate genomic promoter sequences from Ensembl, and tests them using MATCH [82] for the presence of TF binding predictions (matrices) in the transcriptional regulatory database Transfac [26]. In the present work, PAINT was used to retrieve promoter sequences 1000 bp upstream from the transcriptional start sites of the genes on our arrays and search for TF binding sites on both positive and negative strands at a core similarity threshold of 0.9. This upstream length was chosen because the vast majority of known TF binding sites occur within this range [80, 27]. PAINT retrieved promoter sequences and identified at least one known TF binding site prediction for 63% of the genes on our arrays. TF binding predictions that were found more than once in individual gene promoters were treated as individual occurrences. The TF binding site predictions were then grouped according to TF binding site families as defined by Transfac (as opposed to individual binding site matrices). As with GO terms, TF binding site families that were highly correlated with one another in terms of gene promoters (Jaccard similarity > 75%) were agglomerated. Finally, those TF binding site families that occurred in less than five gene promoters on the arrays were filtered out, leaving 162 TF binding site families for subsequent analysis. On average, members of six predicted TF binding site families were found in each gene promoter.

*TF binding attributes: predictions obtained from phylogenetic conservation*

In addition to matching rat promoter sequences directly to TF bnding site predictions from the TRANSFAC database with PAINT, we used TF binding site predictions based on phylogenetic conservation as reported in [27], a comprehensive survey of conserved TF binding site predictions in promoters across four mammals (human, rat, mouse, and dog). They extracted 4000 bp of human promoter sequences centered at the transcription start site of RefSeq genes, performed alignments to the other three genomes, and provided a database of the aligned sequences and the occurrences of the TRANSFAC binding sites in them [27]. Because this database consists of conserved TF binding sites detected in alignments of human sequences to rat, mouse and dog sequences (as opposed to alignments of rat sequences to human, mouse, and dog sequences) and is reported in terms of human genes, it was necessary to map them to the rat genes on our microarrays. Mapping was accomplished using the Homologene database [37] and the annotation tool SOURCE [83]. Approximately 78% of the genes on the arrays could be mapped through Homologene, and 94% of those could be mapped to genes in the database of [27]. Thus TF binding site predictions for 73% of the genes on our arrays were obtained in this manner. As for the PAINT predictions, multiple occurrences of the same binding site prediction in individual gene promoters were treated no differently than single occurrences, TF binding site predictions that were highly correlated with one another (Jaccard similarity > 90%) were agglomerated, and binding sites that appeared in promoters of less than 5 genes on our arrays were excluded Ultimately, 402 individual TF binding site predictions (matrices) remained for subsequent analysis.

*TF binding attributes: predictions using protein-DNA interaction data*

Transcriptional regulatory network analysis is not restricted to TF binding site predictions. When available, genome-wide protein-DNA interaction data can be used in exactly the same manner as binding site predictions, skipping promoter sequences entirely. While genome-wide protein interaction data are not as extensive for mammalian systems as they are for yeast [84], two recent studies provide system-wide DNA binding activities in mammalian tissues for the TFs HNF1-alpha, HNF4-alpha, and HNF6 [29]; and CREB [28]. While neither of the studies considered neuronal tissues, moderate overlap between the TF-gene interactions identified and those in the SCN may be expected. The significance cutoffs employed in the original studies to define the presence or absence of protein-DNA interactions were employed presently. Gene targets of the HNF transcription factors were defined as those that had binding ratios with p-values ≤ 0.001 or fold changes ≥ 2 in either the pancreatic islets or hepatocytes. This gave 2.4% of genes as HNF6 targets, 18% as HNF4-alpha targets, and 2.2% of genes as HNF1-alpha targets. For CREB, two sets of targets were defined, a “strict set”, for which the binding ratio had p ≤ 0.001 and fold changes ≥ 2 for all conditions examined (hepatocytes, pancreatic islets, HEK293 cells, for CREB and phospho-CREB), and a “relaxed set”, for which for which the binding ratio had p ≤ 0.001 for at least one condition and had fold changes ≥ 2 in at least one condition. This gave 0.5% and 21% of genes as strict and relaxed targets of CREB, respectively. As with the database in [27], the protein-DNA interaction data were in terms of human genes, and thus SOURCE [83] and Homologene [37] were used to map the data to homologous rat genes. Using this method, transcription factor-gene links for about 67% of the genes on our microarrays were obtained. There were no strong correlations between any of the five transcription factors (counting relaxed CREB and strict CREB sites separately), with the maximum Jaccard similarity being 39%.

***Meta-analyses***

As mentioned in the main text, gene group enrichments can depend nonlinearly on parameters that define significantly differentially expressed gene groups [25] and microarray results can depend on the normalization approach employed [54]. To identify robust enrichments, we supplemented the gene group enrichment analyses with local and global meta-analysis p-values (p_M_^(LOCAL)^ and p_M_^(GLOBAL)^, respectively).

The local meta-analysis p-value (p_M_^(LOCAL)^) addressed variation in enrichment results obtained when different cutoffs for statistically significant gene expression effects were used at a single normalization (the standard normalization) – and was thus local in normalization space. We computed p_M_^(LOCAL)^ as the geometric mean (GM) of the enrichment p-values (p_ENRICH_) computed for gene groups defined using three cutoffs for significance (FDR < 5%, 2%, and 1%) for the standard normalization for GO and TF binding enrichments. For circadian gene expression enrichments, we used gene group significance cutoffs of (FDR < 20%, 10%, and 5%). The GM of the p-values was used instead of the arithmetic mean so that highly significant enrichments (very low p^ENRICH^) for one or two significance cutoffs would not be overly-penalized. It is possible that at some significance cutoffs there will be less than 5 genes in a particular gene group (for example, only 3 genes regulated by EGF specifically during the day). In this case, p_M_^(LOCAL)^ was the GM of the enrichment p-values computed for gene groups consisting of five genes or more. The motivation for this was to not penalize enrichment results simply because there were too few members of a particular gene group at strict significance cutoffs.

The global meta-analysis p-value (p_M_^(GLOBAL)^) was used to address variation in gene group enrichment results that arises when different significance cutoffs and different normalization approaches are used. The normalization approaches considered encompass all permutations of three independent normalization approaches: (1) whether random-effects ANOVA or fixed-effects [85] ANOVA is used to remove systematic slide and subarray variation, (2) whether or not subarray variance standardization is employed (following array variance standardization employed in [54, 86], and (3) whether the vector normalized Cy5/Cy5 (experimental/vector reference) ratios [49] are used as raw data or simply the Cy5 (experimental) data is used as raw data. It follows that a total of 24 conditions were considered, for the 8 possible normalizations and 3 significance cutoffs, in the global meta-analysis. As for the local meta-analysis, p_M_^(GLOBAL)^ was computed as the GM of enrichment p-values for all conditions under which the particular gene group had 5 or more members. Computation of p_M_^(LOCAL)^ and p_M_^(GLOBAL)^ allowed enrichment results that were robust – and therefore potentially most relevant biologically – to be prioritized for validation.

***qRT-PCR experiments & analysis***

Gene expression levels across the experimental conditions of TFs implicated by the gene group enrichment results (*c-Jun, c-Ets1, Creb1, C/EBPα, C/EBPβ, C/EBPγ,* and *Rorα*) were measured using qRT-PCR. *Fyn* *proto-oncogene* was selected from the microarray data for use as a housekeeping gene on the basis that two independent clones for *Fyn* (UI-R-C1-li-d-11-0-UI, UI-R-E1-fo-e-08-0-UI) had no statistically significant EGF, circadian time, or EGF:CT effects and they had relatively small experimental variability. Additionally, *Fyn* is a relatively long gene (about 200,000 bp, *http://genome.ucsc.edu*), and would require ~2 hrs before changes in transcription initiation would be reflected at the mRNA level. Since the measurements are taken 60 minutes after EGF treatment, any EGF-induced transcriptional changes in *Fyn* would not be observed, although post-transcriptional regulation of *Fyn* mRNA is possible. It is nevertheless important to note that although no literature evidence for differential *Fyn* expression in response to EGFR activation could be found, *Fyn* expression levels have been induced within 8 hrs after brain seizures in some brain regions, and moderately down-regulated (20%) within 1 hr of seizures in others [87].

Amplified RNA obtained from the samples used on the microarrays was used for the qRT-PCR experiments. In addition, two daytime samples (EGF and control) from a fifth rat that were not used in the microarray study were put through two rounds of aRNA amplification and used for qRT-PCR. Amplified RNA quality for qRT-PCR was assessed using Bioanalyzer Picochip (Agilent Technologies, Palo Alto, CA). Reverse transcription (RT) was carried out for 150ng aRNA for each sample in 25 μL reactions (SuperScript II, Invitrogen). The RT product was diluted to 1ng/µL. SYBR Green I (Applied Biosystems, Inc.) was used as a fluorescent reporter in the qRT-PCR reactions. For *c-Ets1* and *Fyn*, qRT-PCR reactions were carried out using 2ng of RT product in 25 µL reactions. Two separate 96 well plates were used, with the ordering of the samples being randomized between the plates. For *C/EBPα*, *C/EBPβ*, *C/EBPγ*, *Rorα*, and *Fyn*, reactions were carried out using 2ng of RT product in 25 µL reactions in triplicate on individual 96 well plates for each TF/housekeeping gene pairing. Reactions were performed using an ABI Prism 7000 device (45 cycles of 1:15 min @ 60°C/ 0:15 min @ 95°C), and amplification curves were obtained using ABI Prism 7000 SDS software. For *Creb1*, *c-Jun*, *c-Ets1*, and *Fyn*, additional reactions were carried out in duplicate for each sample using 0.8ng of RT product in 10 μL reactions in separate quadrants of a single 384-well plate through the TJU qRT-PCR facility.

*qRT-PCR primers*

*Fyn*-Forward: GAAACCACCAAAGGTGCCTA

*Fyn*-Reverse: GCTGCTGAAGGGTCTCAAAC

*c-Jun*-Forward: CTGCAAAGATGGAAACGACCT

*c-Jun*-Reverse: AGCCGTAGGCGCCACTCT

*c-Ets1*-Forward: TTGCGCCATGGAAACCA

*c-Ets1*-Reverse: AACCCAGAGTGTTGATCTCCAAA

*Creb1*-Forward: CCAAACTAGCAGTGGGCAGT

*Creb1*-Reverse: GAATGGTAGTACCCGGCTGA

*C/EBPα*-Forward: AGCAGAAGGTGTTGGAGTTG

*C/EBPα*-Reverse: CTTGACCAAGGAGCTCTCAG

*C/EBPβ*-Forward: GATGCAATCCGGATCAAAC

*C/EBPβ*-Reverse: CCGCAGGAACATCTTTAAGT

*C/EBPγ*-Forward: CTTTTCCCCCTTGTTTGTTT

*C/EBPγ*-Reverse: CACGGTGGCTACTGTCTTTT

*Rorα*-Forward: CGTTTGCACCTTTTTGTTTT

*Rorα*-Reverse: TCCAAGCCTTCTCAGCTAAA

*Rorβ*-Forward: TACGTATCCATGCACTGTGG

*Rorβ*-Reverse: TGAAAACTCTGGCTGGAAAC

*Testing for significant correlations between TFs and EGF-responsive gene profiles*

Previous studies have suggested that expression profile correlations may be indicative of functional regulatory relationships TF and their target genes [47]. While we have demonstrated that gene dynamics may lead to more complex relationships between TF and target expression patterns in some conditions [48], we nevertheless undertook an analysis to test for significant correlations between the TFs implicated by our gene group enrichment analyses and our EGF responsive gene groups. We wanted to determine whether the *overall* correlations between the TF expression profiles and the EGF responsive gene groups was greater than the correlations between the TFs and random gene groups of the same size. We first generated mouse-independent spot-averaged or repeat-averaged expression profiles by subtracting the random rat components estimated by the mixed-model ANOVA of the qRT-PCR (normalized Δ*Cyt*) and microarray (log_2_ expression) data for the TFs and expression profiles, respectively. This ensured that we computed correlations in terms of EGF responsiveness and not animal-animal variability. For each TF and each EGF responsive gene group, we then computed |R|_GROUP_, the average absolute Pearson correlation between the expression profiles of the TF and the genes in the gene group. We used absolute correlation coefficients to count strong correlations and strong anti-correlations between the TFs and target genes with equal weighting. To determine the statistical significance of each |R|_GROUP_ value, we constructed empirical distributions of |R|_RAND_ values, which were |R|_GROUP_ values computed for random gene groups of the same size selected without replacement. P-values were then computed as:

p(|R|) = (# |R|^RAND^ ≥ |R|_GROUP_)/(1 + # |R|_RAND_ computed) Equation S5

which gives the probability of having observed a value of |R|_GROUP_ or better by chance. We performed 2,000 random samplings, giving a minimum possible p(|R|) of 5×10^-4^. We computed these p-values for gene groups defined at significance cutoffs of FDR < 2%.

# List of abbreviations used

ANOVA – analysis of variance

CT – circadian time

EGF – epidermal growth factor

EGFR – epidermal growth factor receptor

GM – geometric mean

GO – gene ontology

ML – maximum likelihood

REML – restricted maximum likelihood

SCN – suprachiasmatic nuclei

TF – transcription factor
