# Supplementary material for: Systems analysis of circadian time-dependent neuronal epidermal growth factor receptor signaling
Source: Genome Biol. 2006 Jun 19;7(6):R48. doi: 10.1186/gb-2006-7-6-r48 (PMC1779538; doi:10.1186/gb-2006-7-6-r48)
Supplement: Additional data file 6 — Additional results. [file gb-2006-7-6-r48-S6.doc]

**Supporting Information: Results**

Focusing specifically on genes we found most strongly regulated by EGFR activation in the SCN revealed several known to be involved in EGFR responses in other systems. *Kdr* (*p^EGF^* = 9×10^-6^, *p^EGF:Clock^* = 1×10^-3^) is a receptor for vascular endothelial growth factor (VEGF) and several studies in other cell types report the induction of VEGF downstream of EGFR [63], as well as the induction of EGFR downstream of VEGF [64]. Strong, night–specific induction of *Kdr* downstream of EGFR observed presently may complete this circuit of co–activation. CamkII is involved in EGF induced gene regulation in other tissue types [65] and the consistent induction of *CamkIIb* observed presently (*p^EGF^* = 5×10^-6^, *p^EGF:Clock^* = 2×10^-1^) may reflect this activity. *Tsc2* (*p^EGF^* = 6×10^-6^, *p^EGF:Clock^* = 1×10^-4^) has been implicated in EGF induced Akt activation [66] and is phosphorylated and degraded upon EGF activation in an Akt–dependent manner in PC–12 cells [67]. The transcriptional regulation of *CamkIIb* and *Tsc2* suggests modulation of EGFR signaling pathways by EGFR activation in the SCN. Induction of both *S100a10* (*p^EGF^* = 2×10^-5^, *p^EGF:Clock^* = 4×10^-5^) and *Cdc25b* (*p^EGF^* = 4×10^-6^,  *p^EGF:Clock^* = 1×10^-5^) by EGF have been reported in other cell types [[68, 69], respectively]. *Cabin1* (*p^EGF^* = 7×10^-7^, *p^EGF:Clock^* = 2×10^-5^) is an inhibitor of calcineurin[70], a phosphatase for the TF Elk–1 [71], which is activated by EGF [72]. Strong down–regulation of *Cabin1* during the circadian night may serve to terminate EGF–induced Elk–1 activity. Strong daytime specific induction of *Scarb1* (*p^EGF^* = 5×10^-6^, *p^EGF:Clock^* = 1×10^-4^) is consistent with previous work showing that combined treatment of muscle cells with growth factors synergistically increases scavenger receptor activity [73]. This clock-dependent differential regulation of several known EGFR-signaling-related genes highlights the importance of circadian context to the EGFR regulated processes in SCN.
